# Educational videos in genetic counseling: Meeting patients where they are?

**DOI:** 10.1016/j.gimo.2025.103436

**Published:** 2025-05-19

**Authors:** Julia Mahal, Carlotta J. Mayer, Sebastian Sailer, Melanie Wittenberg-Marangione, Johanna Tecklenburg, Elena S. Doll, Elias Staatz, Seraina P. Lerch, Hannah Wallaschek, Christian P. Schaaf, Beate Ditzen

**Affiliations:** 1 Institute of Medical Psychology, Center for Psychosocial Medicine, Heidelberg University Hospital, Ruprecht-Karls University Heidelberg, Heidelberg, Germany; 2 Institute of Human Genetics, Heidelberg University Hospital, Heidelberg, Germany; 3 Department of Human Genetics, Hannover Medical School, Hannover, Germany; 4 Department of Medical Psychology, University Medicine Greifswald, Greifswald, Germany

**Keywords:** Educational videos, Genetic consultation, Patient satisfaction, Physician-patient interaction, Physician workload

## Abstract

**Purpose:**

This study examined the effects of self-developed educational videos on patient satisfaction and the physician-patient relationship in genetic outpatient clinics.

**Methods:**

Patients with suspected hereditary breast and ovarian cancer (HBOC, *n* = 311) or requiring exome sequencing for syndromic conditions (SYN, *n* = 52) were randomized to experimental or control groups. Before their consultations, the experimental group viewed videos detailing relevant genetic information. Patient and physician satisfaction and the quality of the physician-patient interaction were then assessed using questionnaires.

**Results:**

The videos were found to be understandable and useful. The video intervention did not affect patient or physician satisfaction scales or the physicians’ workload. However, there were significant interaction effects: The video intervention was associated with significantly lower satisfaction scores among patients with SYN. Additionally, in post-hoc tests, we partially found lower satisfaction with patient decision making, as well as increased physician workload in the SYN group compared with the HBOC group.

**Conclusion:**

Educational videos can improve patient satisfaction in HBOC consultations. In complex and less homogenous clinical situations, such as genetic consultation and testing for syndromic conditions, however, face-to-face physician-patient contact may be more appropriate to reduce stress and adequately address individual questions.

## Introduction

Over the past few decades, the relevance and availability of genomic data in patient care has steadily increased. This is not only due to technological advances that have expanded the repertoire of what is possible but also because the use and processing of genomic data has become more extensive and complex. This has led to an increased need for genetic consultation in the context of genetic testing.^1^ A typical consultation session is time consuming and lasts approximately between 30 and 90 minutes,^2^^,^^3^ during which the patient is provided with a wide range of information, such as the relevance of genetic analyses for other family members, family planning, explanation of the methods used and their limitations, the possibility of secondary findings, potentially negative effects on future insurance, and data privacy.

In general, patients often require information before a medical consultation. As previously observed by Attfield et al,^4^ patients who seek information in advance tend to desire the ability to provide more valuable input and to adopt a more inquisitive stance during the consultation. However, the individual understanding of genetics is highly variable between patients, and there is a need for information at varying levels of depth. In face-to-face interviews, McKibbin et al^5^ identified that many patients desire information regarding their medical diagnosis and the procedure of the genetic testing. This includes knowledge of the implications of the test, the specific elements being tested for, and the estimated time frame for the procedure. Quinn et al^6^ found that one of the highest information needs of patients with rare diseases is related to “basic genetics,” but over 50% of the patients in the study struggled to obtain information in this area. Their top source of information was “internet research,” with “doctors/specialists” ranking as second place.

Recently, educational videos have gained attention as a potential way to provide needed information to patients in many areas of medicine. In genetics, Rana et al^7^ compared video education with traditional genetic consultation for genetic testing in patients with prostate cancer. There was no significant difference in agreement to genetic testing between the group receiving video education only and the group receiving genetic consultation. However, only 1% of those who watched the videos felt the need to talk to a genetic counselor. In terms of satisfaction, the only difference between the 2 groups was that fewer patients who watched the videos felt that all their questions and concerns had been answered compared with those who received genetic consultation. In a similar project conducted by Russo et al,^8^ men with prostate cancer were given the choice of genetic consultation or video education. They reported that 71% of men chose video education over traditional genetic consultation. Satisfaction with the process was comparable between the 2 groups. Similar results were reported by Chavarri Guerra et al,^9^ who showed noninferiority of educational videos compared with classical genetic consultation in patients with suspected cancer predisposition.

In a recent study, Cragun et al^10^ investigated the efficacy of a web-based, animated video tool in the context of genetic consultation for patients with inherited cancer risk. The video, which had a duration of 12 minutes and utilized animations, permitted patients to view and review the content at their own pace. The study demonstrated that the video tool markedly enhanced patients’ knowledge before consultations, particularly regarding standardized cases, such as cancer predispositions, and increased the number of patients willing to undergo genetic testing. Similarly, Denny et al^11^ investigated the efficacy of a 5-minute educational video for patients hospitalized with acute ischemic stroke and intracerebral hemorrhage. The video addressed the recognition of stroke symptoms, the identification of risk factors, and the implementation of prevention strategies. The study demonstrated that the video improved stroke knowledge, self-efficacy in recognizing symptoms, and patient satisfaction with stroke education. Furthermore, these improvements were sustained for a period of 30 days after discharge.

Additional studies suggest an increase in patient satisfaction when patients are shown educational videos. Monteiro Grilo et al^12^ conducted a systematic review and meta-analysis of the effect of educational videos in vascular or imaging procedures: most studies showed a significant improvement in patient anxiety, satisfaction, and efficacy compared with other forms of information. Significant improvements in patient satisfaction have also been demonstrated with video education in candidates for gastroscopy^13^ and for venous thromboembolism education.^14^ In addition, transparent communication through video education has changed the relationship between patients and their physicians. Health education, in general, through various channels, including educational videos, increases patients’ trust in physicians.^15^

In addition to clarifying findings and treatments, the information provided should facilitate patient’ ability to make well-informed decisions. Nevertheless, studies examining the influence of educational videos on patient decision making have yielded inconclusive results. As demonstrated by Chiou and Chung,^16^ watching educational videos has been shown to reduce decision regret among dialysis patients. In contrast, Wilkins et al^17^ found no significant intervention effect when analyzing the impact of informational and decisional preferences of an educational video on patient decision making in breast cancer patients.

A crucial factor that likely influences the efficacy of educational videos is an individual’s attitude toward new technologies. Individuals who exhibit a higher level of technology commitment tend to experience reduced stress when interacting with technology in their daily lives.^18^ Although technology commitment typically reduces stress when utilizing technology, this phenomenon may not manifest consistently across all educational settings. For example, research conducted by Hernan et al^19^ in the field of genetics education revealed that video-based learning, in contrast to expectations, resulted in a reduction of genetic knowledge. This indicates that the efficacy of educational videos may fluctuate, contingent on the intricacy of the subject matter being conveyed. The efficacy of these videos may be dependent on the health literacy of the patient population, with higher levels of health literacy potentially facilitating comprehension and lower levels posing a challenge.^20^

As discussed above, videos have already been used to (partially) replace genetic consultation.^7^, ^8^, ^9^ We did not find any study that evaluated the impact of videos on the workload of physicians when integrated with traditional genetic consultation. However, Hernan et al^19^ investigated the effect of educational videos covering the topic of exome sequencing on the consultation of genetic counselors. Even after the patients have seen the educational videos, the genetic counselors rarely skipped any parts of genetics education, and the time spent with consultation did not differ between patients that had watched the videos and patients that did not watch them. When looking at other fields of medicine, Ihrig et al^21^ compared multimedia-based preoperative consultation (including illustrations, short videos, and written information) with standard consultation. For their consultation, physicians themselves preferred multimedia-based education because they saw this as more understandable, visually appealing, and easy to use when explaining complex issues. Although the physicians had the impression that the effect of multimedia education lasted longer, there was no significant difference in objective time measures.

The impact of educational videos, when integrated into genetic consultation as a supplementary resource rather than a substitute, has yet to be thoroughly examined within a clinical population. Additionally, the extent to which patients with diverse conditions may respond differently to educational videos remains largely unexplored. To ensure the effective utilization of educational videos in a clinical context, it is essential to identify suitable patient groups and ascertain whether these videos can effectively reduce the workload of physicians.

The objective of our study was 2-fold:

1. To develop brief educational videos containing information that is frequently discussed during genetic consultations and to identify which patient groups would benefit most from such educational videos.
2. To evaluate the effect of these videos based on the following hypotheses:
2.1. Patients who have viewed the videos demonstrate greater satisfaction with the genetic consultation and the decisions made during the consultation.
2.2. Patient satisfaction with consultation is dependent upon their general avoidance of information and the extent to which they have previously informed themselves about the subject matter.
2.3. Physicians report a lower workload for patients who have previously watched the videos.

## Materials and Methods

Our project was conducted in 2 stages, corresponding with the objectives. Stage 1 was the preliminary study, during which the educational videos were developed and tested in genetic consultation situations. Stage 2 was the main study.

### Development of educational videos (preliminary study)

The first phase of the study was designed to gather key insights into genetic consultation from both the physician and patient perspectives. This information was used to develop educational video sequences designed to add value for both groups. To refine the content of these videos, the study conducted several moderator-facilitated focus groups with participants from both the physician and patient communities. The preliminary study received ethical approval from the Ethics Committee of the Medical Faculty of Heidelberg (study number: S-054/2021).

The focus groups each included 4 patients or patient representatives and 2 physicians from different specialties, namely, gynecology, oncology, pediatric oncology, and pediatric neurology. The patients and patient representatives were individuals living with, or family members and representatives of those affected by conditions such as autism, rare diseases, *BRCA* pathogenic variants, Li-Fraumeni syndrome, or Prader-Willi syndrome. First, the participants were informed that genetic consultation sessions typically need to cover a lot of information in a limited amount of time. They were asked to identify key topics and content that should be included in short educational videos for patients before their consultations to make these sessions more informative. Participants were also encouraged to provide feedback on these topics and suggest any necessary revisions or additions.

After these focus groups, detailed scripts for 5 short videos were developed based on the insights gathered and presented to a second focus group consisting of 3 patient representatives, 3 patients or their relatives, and 2 physicians. This group was tasked with identifying any missing, unclear, or redundant content.

After feedback from the second focus group, a film company was commissioned to produce 5 animated educational videos, each between 2 and 5 minutes in length. The research team played an integral role in overseeing the production process to ensure the accuracy and effectiveness of the content.

### The educational videos developed for this study address the following topics:

1. The process of genetic consultations
2. Consent, data protection, and data used in genetic consultations
3. Hereditary breast cancer predispositions and associated risk calculation programs
4. The methodology of exome analysis
5. AI-based diagnostic tools, specifically automated image analysis

### Evaluation of educational videos (main study)

After the creation of the videos, a survey was conducted in genetic outpatient clinics at the Heidelberg University Hospital and Hannover Medical School to assess the utility of the videos for patients and physicians. The study population consisted of legally capable adults undergoing genetic consultations. All participants were required to read, understand, and sign a consent form outlining the study procedures. The second part of the study also received ethical approval from the Ethics Committee of the Medical Faculty of Heidelberg (S-058/2022).

### Study participants

The study included both physicians and patients. The patients were selected to represent 2 different subgroups: patients with hereditary breast and ovarian cancer syndrome (HBOC) and patients with syndromic conditions (SYN) and their guardians. Patients’ guardians were invited to participate in the study if patients could not complete the questionnaires themselves (eg, because of cognitive impairment or because they were minors). The physicians were all medical geneticists or residents training for specialization in medical genetics. They offered genetic consultation to both of the patients subgroups.

After scheduling an initial human genetics consultation at either study site, patients were contacted by phone or e-mail. The reasons provided by patients who declined to participate in the study included insufficient German-language skills, lack of necessary technological requirements, lack of interest, and concerns about burden or time resources. Ultimately, a total of 608 participants were successfully recruited at the 2 study sites.

Of the 363 participants with complete data sets, 311 were patients with HBOC, and 52 were patients with SYN. Of the 52 patients with SYN, 16 were index patients and the parents of the remaining minors completed the questionnaires. A total of 186 were assigned to the control group and 177 to the experimental group. A total of 35 physicians conducted the genetic consultation sessions.

### Inclusion and exclusion criteria

Participants were excluded if they received genetic consultation remotely via online platforms or video calls or had language barriers that impeded their understanding of the video content. The study focused only on initial consultations and excluded patients who attended follow-up sessions to discuss results.

### Study procedure and materials

Patients scheduled for initial genetic consultation for syndromic conditions and breast cancer during the study period were invited to participate. After providing informed consent, they were randomly assigned to either the experimental or control group, with the experimental group receiving educational videos in addition to standard consultation and the control group receiving standard consultation only.

**Figure 1:**
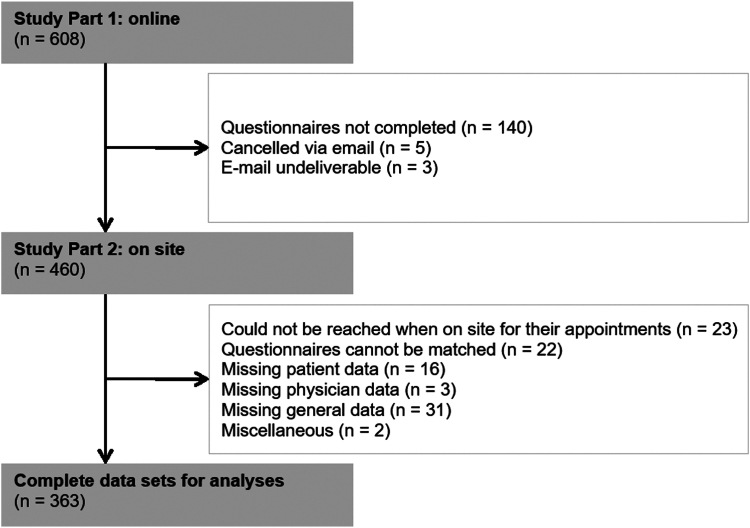
**Flow chart of participant recruitment**.

Before their appointments, all participants completed an online preconsultation survey (part 1, preconsultation). During the preconsultation survey, the HBOC group watched videos 1, 2, and 3, whereas the SYN group watched videos 1, 2, 4, and 5. By sending part 1 approximately 2 weeks before the genetic consultation appointment, participants in the experimental group were able to view the videos from home at a time of their choosing. To ensure that patients had, indeed, viewed the videos, they were required to confirm after the video presentation, that they had watched the videos without experiencing any technical or other disturbances. Patients had the opportunity to view the videos multiple times. We did not trace how often the videos had been watched. After the genetic consultation session, patients and physicians completed several questionnaires (part 2, after consultation) on site. The flow chart in Figure 1 shows the participants in the 2 parts of the main study. Part 1 refers to the online survey before the genetic consultation session, during which the experimental group was shown the videos. Part 2 of the study refers to the postgenetic consultation evaluations with patients and physicians on site (after consultation). For this study, 363 physician-patient data pairs were analyzed.

**Figure 2:**
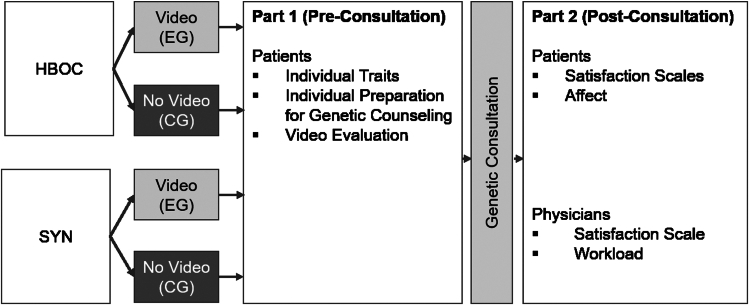
**Study procedure of the main study**. Separation according to patient group and experimental group. Detailed information concerning questionnaires are in the following parts below. CG, control group; EG, experimental group; HBOC, hereditary breast and ovarian cancer; SYN, patients with syndromic conditions.

The study procedure of the main study is detailed in Figure 2.

### Questionnaires

Patient questionnaires (part 1, before consultation)

### Individual traits

To evaluate the patients’ technological commitment, they were asked to indicate their level of agreement with the 12 statements of the Technology Commitment Questionnaire.^22^ Four of the questions pertained to technology acceptance (eg, “I am quick to like new technological developments.”), technology control (eg, “My success in using modern technology depends largely on me.”), and technology competency (eg, “I am often afraid of failing when dealing with modern technology.”). Patients were presented with a 5-point Likert scale, ranging from 1 (indicating “not true at all”) to 5 (indicating “absolutely true”). The questionnaire has been shown to demonstrate high internal consistency, with alpha coefficients ranging from 0.74 to 0.84 for the respective scales.^22^

Information avoidance, defined as the tendency to avoid potentially unwanted information,^23^ was assessed with the Information Avoidance Scale.^24^ The scale comprises 8 items (eg, “I can envisage circumstances in which I would prefer not to be aware of _____.”) that can be adapted to suit particular contexts. Accordingly, to align the questions with the context of this study, the blanks were replaced with a description of the genetic consultation context. Participants were requested to indicate their level of agreement on a 7-point Likert scale, ranging from 1 (indicating “strongly disagree”) to 7 (indicating “strongly agree”). The questionnaire is considered to be reliable and valid.^24^

To ascertain the participants’ health literacy or their need for support for comprehending medical texts, respectively, they were presented with the following item: “How often do you require assistance when reading instructions, pamphlets, or other written material from your doctor or pharmacy?”^20^ Responses were recorded on a 5-point Likert scale ranging from 1 (indicating “never”) to 5 (indicating “always”).

### Individual preparation for genetic consultation

In order to assess the extent to which the patients had informed themselves independently, they were asked to answer the questions listed in Supplemental Information A 2 weeks before the appointment. The questions concerned the duration of preparation by the patients and their assessment of having been sufficiently informed. Preparation was not further specified; therefore, it could encompass both gathering necessary information for the genetic consultation and conducting personal research on relevant topics.

### Video evaluation

Additionally, Supplemental Information A comprises the 3 questions presented to the experimental group after their viewing of the videos. The questions inquired as to the helpfulness, understandability, and quantity of information conveyed in the videos. Ratings of understandability and helpfulness could be indicated on a visual analog scale, with 0 indicating a lack of understanding or helpfulness and 100 indicating the opposite. To ascertain the quantity of information conveyed in the videos, respondents were presented with the options “too much,” “just right,” and “too little.”

Patient questionnaires (part 2, after consultation)

### Satisfaction scales

To assess patients’ satisfaction with the genetic consultation session, they were asked to respond to all 6 items of the Genetic Counseling Satisfaction Scale.^25^ For each item (eg, “My genetic counselor helped me identify what I needed to know to make decisions about what would happen to me”), patients were asked to indicate their level of agreement on a 5-point Likert scale ranging from 1 (indicating “strongly disagree”) to 5 (indicating “strongly agree”).

To assess satisfaction with the decision resulting from the consultation, patients were asked to complete the Satisfaction with Decision Scale.^26^ On 6 items (eg, “I am satisfied with my decision.”), patients could respond on a 5-point Likert scale ranging from 1 (indicating “strongly disagree”) to 5 (indicating “strongly agree”). An evaluation of this scale demonstrated good psychometric properties in terms of both validity and reliability.^26^

To measure the overall satisfaction of both patients and physicians, the Patient Satisfaction Questionnaire was administered to participants.^27^ The questionnaire consists of 5 questions formulated either from the patient’s perspective (PSQ patients) or the physician’s perspective (PSQ physicians), eg, “How satisfied are you with the emotional support you provided to this patient?” The questions are answered immediately after the consultation using a 5-point Likert scale, with responses ranging from “not at all” to “extremely good.” The internal reliability, as measured by Cronbach’s alpha, is α = 0.9 for the PSQ patients and α = 0.87 for the PSQ physicians.^27^

More precisely and beyond satisfaction, the quality of the physician-patient interaction was measured by administering the Physician-Patient Interaction Questionnaire (FAPI^28^) to the patients. This questionnaire consists of 14 items (eg, “The physician gave me enough opportunities to describe my difficulties and problems.”) that are rated on a 5-point Likert scale ranging from 1 (indicating “does not apply”) to 5 (indicating “fully applies”). The questionnaire has demonstrated excellent reliability and validity in an evaluation study.^28^

### Affect

The German version of the validated Positive and Negative Affect Schedule (PANAS^29^) was used to assess the patients’ affective state. Patients were asked to indicate the extent to which 20 adjectives describing positive and negative affective states (eg, proud, scared) applied to them. Patients were asked to select one of 5 response categories. The response options were as follows: 1 (indicating “not at all”), 2 (indicating “a little”), 3 (indicating “moderately”), 4 (indicating “quite a lot,” and 5 (indicating “extremely”). Two dimensions were measured: 1 for positive affective states (PANAS PA) and 1 for negative affective states (PANAS NA).

### Physician questionnaires

As mentioned above, physicians completed the physician version of the Patient Satisfaction Questionnaire (PSQ physicians^27^).

Physician workload was assessed using the German version of the NASA Task Load Index (NASA-TLX), adapted to the medical context.^30^ The physicians were asked to indicate the mental, physical, and temporal demands of the consultation, as well as their performance, ie, success with respect to the goal of the consultation, and their effort and frustration. An evaluation of this questionnaire demonstrated its reliability and validity.^30^

### Statistical analyses

Variables were described using frequencies, means (M), and SDs. Statistical significance was considered at *P* values < .05. To account for multiple testing, probability values were adjusted using the Benjamini-Hochberg method to control the false discovery rate. Descriptive analyses, regression analyses, independent sample *t* tests and 2-way analysis of variances were performed with R (version 4.3.1).

## Results

### Sample description

The HBOC group ranged in age from 19 to 84 years (M = 49.98, SD = 13.16). Participants in the SYN group consisted of 16 index patients and 36 parents of minor patients, resulting in age ranging from 23 to 78 years (M = 40.47, SD = 10.94). The HBOC group consisted of 295 (94.86%) women and 16 (5.14%) men, whereas the SYN group had 42 (80.77%) women and 10 (19.23%) men. All participants indicated that they were proficient in German and could understand the consultations and videos.

The M score for how well informed both patient groups felt before genetic consultation was 57.46 (SD = 27.13). The HBOC and SYN groups did not differ significantly in their rating of feeling well informed about genetic consultation when asked before video exposure (*t*(67.94) = 0.15, *P =* .885, d = 0.02; HBOC: M = 57.55, SD = 27.03; SYN: M = 56.94, SD = 27.94). The average duration of the preparation for the genetic consultation session was 78.87 minutes (SD = 99.38) for both groups of patients. The HBOC and SYN groups did not differ significantly in the duration of their preparation for genetic consultation (*t*(75.03) = −1.10, *P* = .275, d = −0.15; HBOC: M = 76.40, SD = 103.42; SYN: M = 91.38, SD = 75.44). The mean health literacy score for both groups was 1.42 (SD = 0.69). There was no significant difference in health literacy scores between the HBOC group (M = 1.42, SD = 0.69) and the SYN group (M = 1.46, SD = 0.73; *t*(67.07) = −0.40, *P =* .688, d = −0.06).

### Video evaluation

For further details regarding the methodology used in the video evaluation process, please refer to Supplemental Information A. The evaluation of the videos was based on the responses of the experimental group that watched them. The participants indicated that they found the videos to be understandable, with ratings ranging from 93.75 to 98.85.

Overall, the videos were rated as helpful. The ratings for the videos ranged from 79.21 to 95.81, with video 2 (“Consent, data protection, and data use in genetic consultations”) receiving the highest rating and video 4 (“Methodology of exome analysis”) receiving the lowest.

Overall, the participants considered the amount of information presented in the videos to be adequate. Results are shown in Table 1. Most responses for each video indicated that the information was just right. However, participants desired more information for videos 4 and 5 (“AI-based diagnostic tools, specifically Face2Gene”) compared with the others.

**Table 1:** Participants’ evaluation of videos’ amount of information

Video	Amount of Information (*n*)		
	Too little	Just right	Too much
1	14	162	0
2	6	168	3
3	4	138	10
4	5	17	2
5	4	20	0

For understandability ratings of video 1, a Welch 2-sample *t* test revealed no significant difference in Ms between the HBOC group (M = 98.18, SD = 11.59) and the SYN group (M = 96.88, SD = 10.41; *t*(32.63) = 0.56, *P =* .577, d = 0.11). Similarly, the analysis of helpfulness ratings of video 1 showed no significant difference between the HBOC group (M = 92.92, SD = 17.72) and the SYN group (M = 83.58, SD = 26.68; *t*(26.28) = 1.66, *P =* .109, d = 0.49).

Regarding the evaluations of video 2, no significant differences were found in the understandability ratings between the HBOC group (M = 98.88, SD = 8.37) and the SYN group (M = 98.63, SD = 6.11; *t*(38) = 0.18, *P =* .857, d = 0.03). Additionally, the analyses for helpfulness ratings of video 2 showed no significant differences between the HBOC group (M = 96.27, SD = 13.60) and the SYN group (M = 92.83, SD = 20.69; *t*(26.21) = 0.79, *P =* .437, d = 0.32). The detailed ratings for understandability and helpfulness are shown in Supplemental Table 1.

### Predictors of video evaluation

Technology commitment, duration of preparation for genetic consultation, and need for information were analyzed as predictors of understandability and helpfulness of all videos. Regression analyses showed that the understandability of the educational videos could not be predicted by technology commitment, duration of preparation for genetic consultation, or need for information. Helpfulness of video 2 was positively associated with technology commitment (*b* = 0.40, *P* = .014) and need for information (*b* = 0.13, *P* = .004; *F*(3,117) = 6.07, *P* = .001, R^2^ = 0.14). Helpfulness of video 4 showed a positive association with the need for information (*b* = 0.99, *P* < .001; *F*(3, 17) = 19.95, *P* < .001, *R*^*2*^ = 0.76). Need for information was positively associated with helpfulness of video 5 (*b* = 0.91, *P < .*001; *F*(3,17) = 18.01, *P < .*001, *R*^*2*^ = 0.76). All other regression analyses were not significant (*P > .*05).

### Health literacy

There was no significant difference in health literacy between HBOC and SYN groups (*t*(67.07) = −0.40, *P =* .688, d = −0.06; HBOC: M = 1.42, SD = 0.69; SYN: M = 1.46, SD = 0.73) and no significant difference between experimental and control groups (*t*(358.62) = 0.32, *P =* .751, d = 0.03; experimental group (EG): M = 1.41, SD = 0.70; CG: M = 1.44, SD = 0.68). Health literacy was negatively correlated with understandability ratings of video 4 (*r* = −.57, *P < .*05). All other correlations between health literacy and video ratings were not significant (*P > .*05).

### Feeling well informed about genetic consultation

An independent samples *t* test was performed to examine differences in scores between the HBOC (M = 57.55, SD = 27.03) and SYN (M = 56.94, SD = 27.94) patient groups. The results indicated no difference between patient groups in their feeling of being well informed about genetic consultation (*t*(67.94) = 0.15, *P =* .885, d = 0.02).

### Group differences

#### Patient variables

##### Genetic counseling satisfaction scale

**Figure 3:**
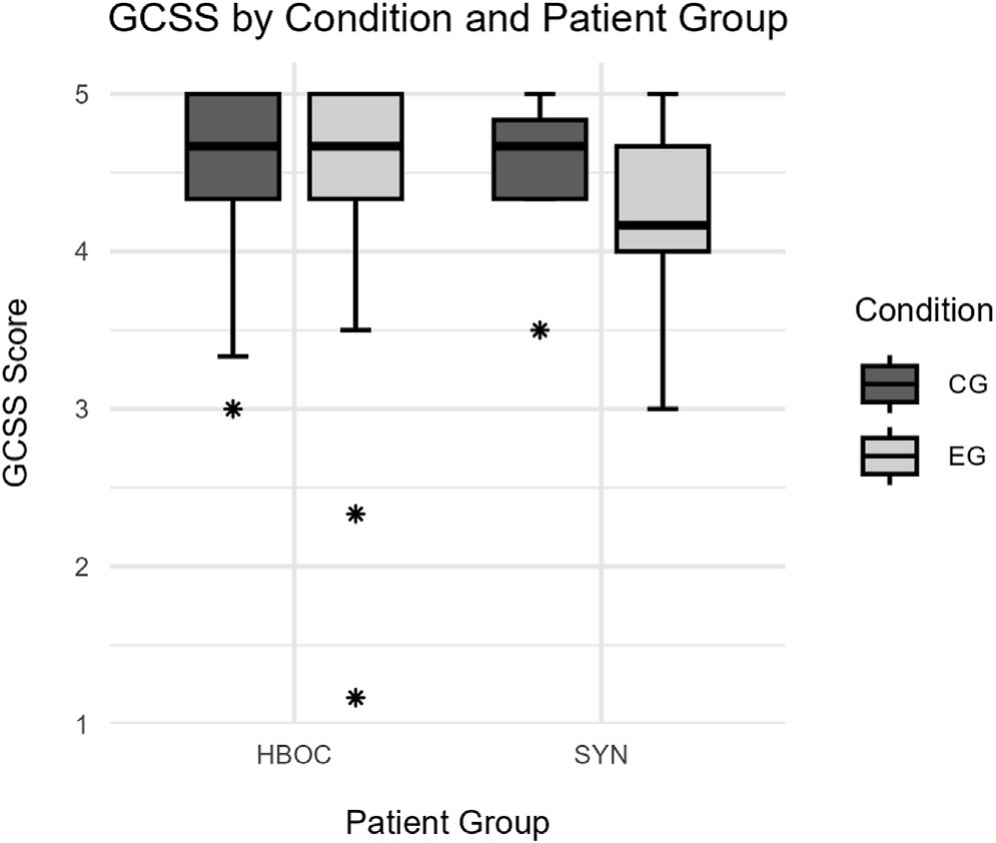
**Genetic Counseling Satisfaction Scale (GCSS) differentiated between patient group and condition.** The boxes represent the interquartile range (IQR) with the median shown as a horizontal line. Whiskers extend to 1.5 times the IQR. Points indicate outliers. Patient groups are those with hereditary breast and ovarian cancer (HBOC) and syndromic conditions (SYN). Conditions are experimental group (EG) and control group (CG).

A 2-way ANOVA for the effects of patient group and condition on the Genetic Counseling Satisfaction Scale (GCSS) revealed a significant main effect of patient group (*F*(1, 355) = 6.38, *P =* .012, d = 0.38; HBOC: M = 4.59, SD = 0.46; SYN: M = 4.41, SD = 0.51) with higher GCSS in patients with HBOC, no significant main effect of condition (*F*(1, 355) = 2.90, *P =* .090, d = 0.17; EG: M = 4.53, SD = 0.53; CG: M = 4.61, SD = 0.40), and a significant interaction between patient group and condition (*F*(1, 355) = 8.39, *P =* .004). Post-hoc Tukey tests with Benjamini-Hochberg correction showed that the SYN group in the experimental group (M = 4.19, SD = 0.57) had significantly lower scores compared with the HBOC group in the control group (M = 4.60, SD = 0.41; *P =* .002, d = −0.96) and the SYN group in the control group (M = 4.62, SD = 0.34; *P =* .011, d = −0.94). The SYN group in the experimental group (M = 4.19, SD = 0.57) also had significantly lower scores than the HBOC group in the experimental group (M = 4.58, SD = 0.51; *P =* .002, d = −0.76). All other pairwise comparisons were not significant (all *P*’s >.998). Figure 3 shows group differences in GCSS.

##### Satisfaction with decision

**Figure 4:**
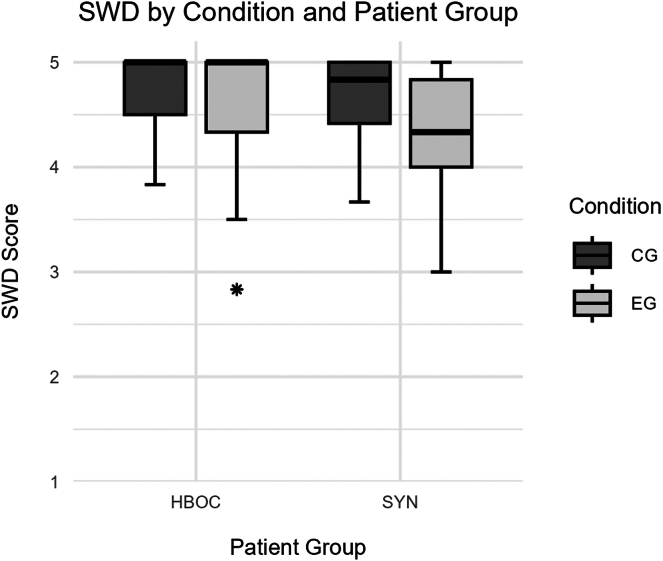
**Satisfaction with decision (SWD) differentiated between patient group and condition.** The boxes represent the interquartile range (IQR) with the median shown as a horizontal line. Whiskers extend to 1.5 times the IQR. Points indicate outliers. Patient groups are those with hereditary breast and ovarian cancer (HBOC) and syndromic conditions (SYN). Conditions are experimental group (EG) and control group (CG).

A 2-way ANOVA for the effects of patient group and condition on Satisfaction with Decision (SWD) scores revealed a significant main effect of patient group (*F*(1, 348) = 8.01, *P =* .005, d = 0.43; HBOC: M = 4.70, SD = 0.43; SYN: M = 4.51, SD = 0.51) with higher scores in the HBOC group, no significant main effect of condition (*F*(1, 348) = 2.51, *P =* .114, d = 0.16; EG: M = 4.63, SD = 0.49; CG: M = 4.70, SD = 0.39), and a significant interaction between patient group and condition (*F*(1, 348) = 4.637, *P =* .032). Post-hoc Tukey tests with Benjamini-Hochberg correction showed that the SYN group in the experimental group (M = 4.33, SD = 0.56) had significantly lower scores compared with the HBOC group in the control group (M = 4.71, SD = 0.39; *P =* .004, d = −0.91). The SYN group in the experimental group also had significantly lower scores than the HBOC group in the experimental group (M = 4.68, SD = 0.47; *P =* .008, d = −0.72). All other pairwise comparisons were not significant, including the SYN group in the experimental group compared with the SYN group in the control group (M = 4.65, SD = 0.41; *P =* .098, d = −0.66), and other comparisons (all *P*’s >.993). Figure 4 shows group differences in SWD by patient group and condition.

##### Physician-patient relationship

The 2-way ANOVA for the effect of patient group and condition on physician-patient relationship (FAPI) revealed a significant interaction effect between patient group and condition (*F*(1, 337) = 4.93, *P =* .027). The main effect of patient group (*F*(1, 337) = 2.87, *P =* .091, d = −0.26; HBOC: M = 4.34, SD = 0.59; SYN: M = 4.49, SD = 0.52) and the main effect of condition were not significant (*F*(1, 337) = 0.01, *P =* .918, d = 0.02; EG: M = 4.35, SD = 0.52; CG: M = 4.36, SD = 0.63). Post-hoc Tukey tests with Benjamini-Hochberg corrected *P* values showed no significant group differences. The SYN group in the control group (M = 4.64, SD = 0.38) had nonsignificantly higher scores than the HBOC group in the control group M = 4.31, SD = 0.6; all *P*’s > .190). The difference between the HBOC group in the experimental (M = 4.36, SD = 0.51) and control groups (M = 4.31, SD = 0.66) was not significant (*P =* 1.000, d = 0.09). Similarly, the difference between the SYN group in the experimental (M = 4.30, SD = 0.61) and control groups (M = 4.64, SD = 0.38) was not significant (*P =* .317, d = −0.69). The difference between the HBOC group in the experimental group (M = 4.36, SD = 0.51) and the SYN group in the control group (M = 4.64, SD = 0.38) was not significant (*P =* .285, d = −0.57).

##### Patient satisfaction scale

The 2-way ANOVA for analyzing the effects of patient group and condition on Patient Satisfaction Scale (PSQ Patients) revealed no significant main effects of patient group (*F*(1, 356) = 0.83, *P =* .364, d = 0.14; HBOC: M = 23.16, SD = 2.72, SYN: M = 22.78, SD = 2.81), or condition (*F*(1, 356) = 0.41, *P =* .522, d = −0.07; EG: M = 23.20, SD = 2.44; control group (CG): M = 23.01, SD = 2.99). However, there was a significant interaction between the patient group and the condition (*F*(1, 356) = 9.24, *P =* .003). Post-hoc Tukey tests with Benjamini-Hochberg correction showed no significant pairwise comparisons (all *P*’s > .152).

##### Positive affect

The 2-way ANOVA to analyze the effects of patient group and condition on Positive Affect scores revealed no significant main effects of patient group (*F*(1, 293) = 0.25, *P =* .616, d = 0.08; HBOC: M = 3.25, SD = 0.56; SYN: M = 3.21, SD = 0.65), or condition (*F*(1, 293) = 0.01, *P =* .930, d = −0.01; EG: M = 3.25, SD = 0.56; CG: M = 3.24, SD = 0.59). There was no significant interaction between the patient group and the condition (*F*(1, 293) = 1.84, *P =* .176). Post-hoc Tukey tests with Benjamini-Hochberg correction showed no significant pairwise comparisons. All combinations within the interaction terms were not statistically significant (all *P*’s >.994).

##### Negative affect

The 2-way ANOVA for analyzing the effects of patient group and condition on negative affect scores revealed a significant main effect of patient group (*F*(1, 303) = 6.08, *P =* .014, d = −0.40; HBOC: M = 1.44, SD = 0.46; SYN: M = 1.63, SD = 0.56), but no significant effect of condition (*F*(1, 303) = 0.72, *P =* .398, d = −0.10; EG: M = 1.49, SD = 0.53; CG: M = 1.44, SD = 0.44). Additionally, there was no significant interaction between the patient group and the condition (*F*(1, 303) = 0.02, *P =* .876). Post-hoc Tukey tests with Benjamini-Hochberg correction revealed that there was a significant difference between the SYN and HBOC groups (*P =* .014, d = −0.40), indicating higher negative affect scores in the SYN group. All other pairwise comparisons, including those within the interaction terms, were not statistically significant (all *P*’s >.740).

#### Physician variables

##### Physician satisfaction scale

A 2-way ANOVA for the effects of the patient group and the condition on Physician Satisfaction Scale (PSQ Physicians) scores revealed no significant main effects, neither for the patient group (*F*(1, 358) = 0.75, *P =* .387, d = 0.13; HBOC: M = 19.89, SD = 3.31; SYN: M = 19.45, SD = 3.79) nor for the condition (*F*(1, 358) = 0.03, *P =* .862, d = 0.01; EG: M = 19.80, SD = 3.39; CG: M = 19.86, SD = 3.38). Additionally, there was no significant interaction between the patient group and the condition (*F*(1, 358) = 0.64, *P* = .424). Post-hoc Tukey tests with Benjamini-Hochberg corrected *P* values indicated that all pairwise comparisons were not significant (all *P*’s >.999).

##### Physician workload

**Figure 5:**
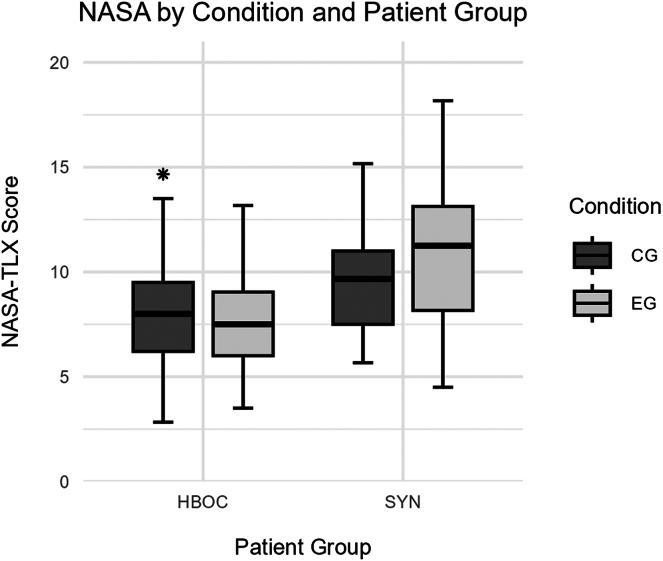
**Physician workload (NASA-TLX) differentiated between patient group and condition.** The boxes represent the interquartile range (IQR) with the median shown as a horizontal line. Whiskers extend to 1.5 times the IQR. Points indicate outliers. Patient groups are patients with hereditary breast and ovarian cancer (HBOC) and patients with syndromic conditions (SYN). Conditions are experimental group (EG) and control group (CG).

A 2-way ANOVA for the effects of patient group and condition on physician workload (NASA-TLX) scores revealed a significant main effect of the patient group (*F*(1, 357) = 45.25, *P <* .001, d = −1.01; HBOC: M = 7.77, SD = 2.25; SYN: M = 10.19, SD = 3.07), no significant main effect of condition (*F*(1, 357) = 0.50, *P =* .480, d = 0.08; EG: M = 8.01, SD = 2.55; SYN: M = 8.21, SD = 2.49), and a nonsignificant interaction between patient group and condition (*F*(1, 357) = 3.31, *P* = .070). Post-hoc Tukey tests with Benjamini-Hochberg correction showed that physicians of the SYN group in the experimental condition (M = 10.69, SD = 3.34) had significantly higher workload scores compared with physicians of the HBOC group in the control group (M = 7.95, SD = 2.35; *P =* .001, d = 1.10) and compared with physicians of the HBOC group in the experimental group (M = 7.59, SD = 2.13; *P <* .001, d = 1.33). Additionally, physicians of the SYN group in the control group (M = 9.74, SD = 2.80) had significantly higher workload scores compared with physicians of the HBOC group in the control group (M = 7.95, SD = 2.35; *P =* .003, d = 0.74). All other pairwise comparisons were not significant (*P =* .539). Figure 5 shows group differences in NASA-TLX scores.

## Discussion

This study examined the impact of educational videos on patient satisfaction and physician workload in genetic consultation. The videos were rated as helpful and understandable, with no significant differences in ratings between the HBOC and SYN groups. Both patient groups reported high levels of satisfaction with consultation, but the SYN group in the experimental group reported reduced satisfaction compared with the control group. Patients with HBOC, however, consistently indicated high satisfaction with their genetic consultation regardless of video exposure. In addition, physicians generally reported less workload with patients with HBOC regardless of experimental condition.

Previous studies have demonstrated significant improvements in patient satisfaction with the use of educational videos, such as those by Monteiro Grilo et al^12^ for vascular or imaging procedures, Kamyabi et al^13^ for gastroscopy candidates, and Marini et al^14^ for venous thromboembolism education. In contrast, our study found no significant positive overall effect of videos on satisfaction in genetic consultation. This suggests that the condition and specific needs of the patient population play a critical role in determining the effectiveness of educational videos on patient satisfaction in the complex context of genetic consultation sessions. It is important to note that the genetic counseling process may differ significantly between the 2 patient groups in this study—HBOC and SYN. Although both groups watched the same introductory video on the “Process of Genetic Consultation” the genetic consultations themselves differ substantially for these 2 conditions. HBOC consultations typically address specific, well-defined genetic conditions and their impact on family members, whereas SYN consultations are more complex and heterogeneous, addressing a broader range of potential genetic diagnoses and more individualized cases. As a result, the video may not be as effective in addressing the specific needs of patients with SYN as it is for patients with HBOC, whose consultations are more focused. However, both groups found the video helpful, suggesting that it effectively provided essential information. Nevertheless, when incorporating educational videos into genetic counseling, it is important to consider the unique needs of each patient group.

When comparing the SYN group with the HBOC group, we found that patients with SYN reported lower satisfaction with genetic consultation and lower satisfaction with their decision after video exposure compared with the HBOC group. This may be due to the different topics discussed in the 2 patient groups: although the diagnostics and implications discussed in the HBOC group were more homogeneous, the SYN group discussed a variety of suspected conditions and implications for their personal and family life. Therefore, although the evaluation of the videos was positive, the videos might not have addressed patients’ needs as thoroughly as needed. All participants in the experimental group watched the genetic consultation and consent videos. In addition, each patient group watched different videos developed for their conditions. As a result, the video material for the HBOC group was more tailored to their specific needs, whereas the SYN group received relatively broad material that did not necessarily meet their information needs. This suggests that complex cases tend to require more personal physician-patient interaction to adequately address their questions. Although satisfaction ratings (GCSS, PSQ Patients, and SWD) were lower for the experimental group of the SYN group than for patients with HBOC, they were nonetheless generally good and remained within an acceptable range. Interestingly, videos 1 and 2, which dealt with practical issues, such as “the process of genetic counselling” and “consent, privacy, and data use in genetic counseling,” were rated as most helpful by both groups. In contrast, videos 4 and 5 were rated as least useful. These videos covered more abstract topics, such as “methodology of exome analysis” and “AI-based diagnostic tools, especially automated image analysis” and were presented to the SYN group only. We thus conclude that educational videos that provide foundational, general information are effective in conveying essential knowledge to patients. However, for more specific clinical situations, a one-size-fits-all approach may be less suitable because patients often have highly individualized needs in these contexts.

In addition, the duration of our videos was relatively short, between 3 and 5 minutes. This time frame may be optimal for conveying practical information, but more complex topics may require a longer, more detailed video to avoid creating confusing or incomplete knowledge among patients.

Regarding the group differences in physicians’ stress and workload, it appears that the overall consultation situation, which involved more individuals (eg, parents with their minor), and less homogeneous topics discussed, had a greater impact on the overall burden than the videos. In line with this, physicians in the SYN group reported higher negative affect, which is likely a consequence of stress.

Second, the SYN group often did not have a suspected genetic diagnosis to discuss; rather they addressed a more vaguely defined condition (eg, intellectual disability) with unclear causal factors. This vagueness may have contributed to ineffective communication on the part of the physicians. In contrast, patients with HBOC were mostly adults and frequently attended the consultation unaccompanied, which may have allowed more focused communication. In the context of HBOC counseling, the set of genes to be discussed is more clearly defined, allowing to focus the consultation on an area of expertise.

Although not statistically significant, the physician workload was found to be higher in patients with HBOC in the control group than in patients with HBOC in the video condition. This indicates a tendency toward reduced physician workload after video exposure in homogeneous patient groups. Physicians were informed in advance that some of their patients may have viewed the educational videos, but they were not told which specific patients had done so. This decision was made to ensure a standardized approach to consultation and to avoid potential bias in physician-patient interactions. Had the physicians known which patients had watched the videos, they might have adjusted their explanations accordingly—either by omitting certain details or by assuming a higher level of baseline knowledge. Although this could have potentially reduced redundancy, it could also have led to inconsistencies in the quality and depth of consultation. In addition, the study results suggest that the videos did not significantly reduce physician workload. One possible explanation is that physicians, not knowing exactly who had watched the videos, provided the same level of explanation to all patients to ensure completeness.

Further research could conduct a study in which the physicians knew which patients had watched the videos beforehand and which had not. In such cases, patients in the experimental group may not have needed additional explanations from the physician, which could have resulted in a reduced physician’s workload. Nevertheless, it seems also likely that patients did not interrupt the physician when information the patient already knew from the videos was presented. Another plausible explanation could be that patients asked more detailed questions after watching the videos, requiring the same amount of physician effort as in the control group. Careful consideration needs to be given to what medical information can be presented exclusively by video without requiring the physician to repeat it as part of the general consultation.

In the online preconsultation survey sent to both the HBOC and SYN groups, patients reported the amount of time they had spent gathering information before their appointment. On average, both groups reported spending about 75 to 90 minutes preparing for their genetic consultation session or their suspected condition, with a wide range of preparation times. This variability suggests that patients have differing information needs and that patients either did not have access to the necessary information or could not prepare adequately because the diagnosis was often not established before the genetic consultation session. This highlights the importance of providing meaningful, understandable, and structured information materials in advance of genetic consultation appointments. Offering a variety of information options could allow patients to select the level of detail and preparation time that best suits their needs. However, our study design, which measured preparation time on a single occasion 2 weeks before the appointment, did not capture any additional preparation that patients may have undertaken in the intervening period. Therefore, we cannot determine the extent to which preparation time may have influenced patients’ satisfaction. Further research could examine the impact of preparation duration on satisfaction and knowledge gain, as well as the specific sources patients utilize for self-education. This could help to refine educational materials.

In addition, specific information about patients’ possible condition is difficult to find for SYN cases, leading patients to seek less relevant or accurate information. Patients who do not want to know more about diseases, genetic testing or diagnostics are generally less satisfied. From the present data, we assume that only patients who actively seek information should receive it: In our study, patients with low information avoidance scores were more satisfied with consultation and decision making. Therefore, additional information should be provided primarily to those who express a desire to know more. The combination of both basic information and videos with basic details, along with the option for more in-depth information, would be beneficial.

Both patient groups rated the videos on general genetics, consent, privacy, and data use as useful, indicating a need for basic understanding and a focus on the ethical and legal aspects of genetic testing. This suggests that patients, regardless of the complexity of their individual cases, not only value structured information on these basic topics but also have a general need to understand them.

### Strengths and limitations

This study has several limitations. Overall, the evaluation demonstrated a ceiling effect, whereby the genetic consultation was rated highly by both patient groups at both sites. As a result, the potential improvement with the video sequences was limited. Several studies have reported high levels of satisfaction with current methods of genetic consultation, which may explain the high levels of satisfaction in our study.^25^^,^^31^, ^32^, ^33^, ^34^ The environment in which participants viewed the videos was not standardized. Thus, the ratings may have been influenced by distractions in the non-standardized environment. It is possible that patients within the SYN group responded differently depending on their suspected genetic condition, creating specific subgroups within the SYN group. This may have masked possible effects in these subgroups. Because of the small sample size of the SYN group, it was not possible to analyze subgroup differences.

In addition, patients and their caregivers in the SYN group may be more likely to be stressed during the consultation sessions than those in the HBOC group, although this was not empirically measured. A possible reason might be that the diagnosis was often not established before the genetic consultation session. Additionally, parents often had to manage the demands of the consultation session while simultaneously attending to their children, who were frequently the index patients. This additional responsibility likely increased their stress levels and may have affected their ability to concentrate fully on the information being provided. The smaller subgroup of patients who are syndromic compared with patients with HBOC also presented a challenge because a larger sample size would have been required to detect smaller effects. However, because patients with SYN are relatively rare compared with patients with HBOC, a larger sample size was not feasible.

Although our study did not directly differentiate within the HBOC group, it is likely that significant heterogeneity occurs, which could influence how the educational videos are perceived and thus contribute to the overall effectiveness of the videos. Patients attended HBOC consultations for various reasons: some were index patients with a personal history of breast or ovarian cancer, whereas others attended predictive testing. This distinction is important because the baseline context and informational needs may vary considerably between these subgroups. For example, index patients, because of their own diagnosis, may have different levels of understanding and expectations for consultation compared with patients who are asymptomatic seeking preventive testing.

The gender distribution was skewed in both groups, with a high proportion of women. This gender distribution was expected in the HBOC group. However, the SYN group was also predominately female (over 80%). An explanation for this could be that minors are often accompanied to medical appointments by their mothers. The very high proportion of women in the study limits the generalizability of the results to men. However, because this study was conducted in a clinical setting with regular patients during their genetic consultation appointments, it can be assumed that the reported results most likely reflect clinical reality.

It is important to note that we focused solely on assessing the quality of genetic consultation through patient satisfaction. We did not evaluate outcomes such as knowledge acquisition, changes in consenting behavior, or changes in perspective on incidental findings. Cragun et al^10^ previously reported an increase in patient knowledge after presenting a video-based online tool to a specific cohort of patients with suspected hereditary cancer predisposition. Future projects could explore similar effects of knowledge on patients with more complex conditions or the effect of knowledge on consenting behavior.

Health literacy was assessed using a single-item measure in this study. This decision was made to balance the need for relevant data with the practical limitations of our study population. Although some patients underwent extensive evaluations as part of their genetic consultation, others had more limited procedures. However, genetic consultation can be a potentially stressful experience because it often involves processing complex medical information and considering important personal and family implications. Given our primary focus on evaluating the effects of educational videos on patient satisfaction and physician workload, we sought to minimize additional burden by avoiding a lengthy health literacy questionnaire.

However, a more detailed assessment of health literacy would be valuable for tailoring educational materials to individual knowledge levels.

In this study, the perspectives of both patients and their physicians were included in the analysis, which is a key strength of this study. Capturing both viewpoints provides a comprehensive understanding of the consultation process, highlighting not only patients’ perceptions and needs but also the challenges physicians face in delivering information. Compared with similar studies, we recruited a large number of participants and collected the data at 2 sites, reducing the influence of site-specific bias in the consultation. The number of participants reached provides a solid basis for drawing reliable conclusions about the data points measured. Notably, the results from the HBOC group provide a robust estimate of the effectiveness of the videos. Our findings indicate that educational videos may be more effective for patient groups with well-defined conditions, such as HBOC, in which structured content can directly address common patient questions. In contrast, for patients with more complex and varied conditions, such as the SYN group, video content alone may not sufficiently meet their information needs. This insight offers valuable guidance for future development of educational videos, emphasizing the importance of aligning video content closely with patient-specific informational requirements.

Although, in this study, the physicians did not know which patients had accessed the videos, knowledge of prior video content would allow them to build on the information that patients had already received. This could enable physicians to focus more on specific patient questions, thereby enhancing the relevance and depth of the consultation. Such an approach acknowledges patients’ substantial preparation time (75-90 minutes on average) and supports a more efficient, patient-centered interaction.

Considering the overall small number of patients with SYN eligible for exome or genome sequencing, the numbers collected can be seen as a good basis to delineate future projects.

We were able to minimize the additional time patients spent in the clinic for the survey by giving them access to the videos at home instead of showing the videos right before the consultation. Immediately after the consultation, patients completed an evaluation of the consultation to avoid possible hindsight bias.

The direct involvement of patient groups in both the development of the videos and the collection of feedback on their usefulness and applicability to everyday medical scenarios represents a strength of this study and the data presented. The videos were developed in collaboration with patients and medical professionals to ensure high-quality information that meets current standards.

To make the videos accessible to a broader audience, English versions of the videos are available in addition to the original German-language videos. The produced videos are freely available for further use in patient education and can be accessed via the following link: https://www.klinikum.uni-heidelberg.de/genki.

### Implications and future research

Our findings suggest that a one-size-fits-all approach to video education may not be suitable for all patient populations but may be useful in standardized situations with frequently asked and clear questions. Video education appears to be less beneficial for patient groups in scenarios involving a broad set of potential genetic diagnoses or rare conditions, in which the uncertainty before a definitive diagnosis makes it difficult to provide tailored, comprehensive information. In these cases, it is difficult to anticipate the specific knowledge patients will need, potentially limiting the effectiveness of prediagnosis educational materials. In such situations, interaction with a health care professional is paramount. To implement educational videos in genetic consultation, videos should be optimized for 2 factors: First, the videos should be appropriate for the patient population and either provide actionable information or answer questions that patients already have. This is easier to implement in highly standardized consultation sessions. Second, the videos should be able to meet individual information needs. This could be achieved by providing short videos that cover the most relevant topics for the upcoming consultation session. Additionally, videos with more detailed information and likely exceeding a duration of 3 to 5 minutes could be provided.

Another interesting question to explore would be the effect on patient satisfaction of providing patients with a choice of videos, with increasing depth of information in the videos. This approach would allow those seeking in-depth details to access longer videos, whereas patients interested only in procedural information could find that in shorter videos. This also includes providing educational videos in simplified language so that people, such as patients with cognitive impairments, can understand their condition and the decisions made during the consultation. Future research could also further explore the use of telemedical services and tailored videos in genetic consultation to provide more comprehensive and personalized support across different patient groups.

The videos took time to develop, involved multiple feedback loops with experts, and were produced by an outside company. Although this process ensured high-quality and validated content, it was time and resource intensive. The videos used in our study were not AI generated but rather carefully scripted and produced based on expert and patient input. However, future advances in video technology may offer significant advantages. More scalable and adaptable approaches could allow for easier updates to reflect new medical guidelines and more tailored content for different patient populations. With a greater volume of educational videos covering highly specific topics, patient education could become more individualized and responsive to different information needs. However, rigorous expert validation would remain critical to ensure factual accuracy and understandability.

Our findings indicate that genetic consultation for patients with SYN was associated with higher physician workload and lower patient satisfaction compared with patients with HBOC, suggesting a more complex consultation process in this group. Given the complexity and ambiguity of many conditions in this group, these patients face unique challenges in preparation and receiving suitable information.

In the future, combining educational videos with telehealth services could provide greater flexibility in the delivery of medical information and support. As telemedicine continues to grow in importance, on-demand access to structured, expert-reviewed video content could help patients receive relevant and up-to-date information at their own pace, regardless of location or scheduling constraints. Although this study did not directly examine telemedicine, future research could explore how integrating telemedicine consultations with tailored educational videos could improve efficiency, reduce physician workload, and better prepare patients for their appointments.

In summary, educational videos can be a valuable adjunct to genetic consultation, particularly for patient groups with well-defined conditions and common concerns. In such cases, videos can help standardize information delivery and potentially reduce some aspects of physician workload. As telemedicine and digital health tools continue to expand, educational videos may play an increasing role in improving the flexibility of patient education. The ability to provide structured, expert-reviewed video content before consultations could help patients better prepare for and engage in more meaningful discussions with their physicians. Future research should explore how best to integrate video-based education with other digital health services to ensure that these tools enhance, rather than replace, the physician-patient relationship.

## Data Availability

The data supporting this research are available from the heiDATA repository at https://doi.org/10.11588/data/HYVQSV. For reviewers, we provided this link https://heidata.uni-heidelberg.de/privateurl.xhtml?token = ceff79d2-9e59-47d7-bbf2-0aa2e759a91c.

## Conflict of Interest

The authors declare no conflicts of interest.

## Additional Information

Supplemental Material A: Self-generated questions Feeling of being informed: How well-informed do you feel about your upcoming appointment in genetics? (0 = not at all, 100 = very good) Video Evaluation Understandability: How understandable were the contents of the video for you? (0 = not understandable at all; 100 = very understandable) Helpfulness: How helpful did you find the video you watched? (VAS, 0 = not helpful at all; 100 = very helpful) Amount of information: The amount of information in the video was…? (too much, just right, too little, (single choice item))
