# Supplementary material for: Educational videos in genetic counseling: Meeting patients where they are?
Source: Genet Med Open. 2025 May 19;3:103436. doi: 10.1016/j.gimo.2025.103436 (PMC12275682; doi:10.1016/j.gimo.2025.103436)
Supplement: Supplemental Material — A: Self-generated questions Feeling of being informed: How well-informed do you feel about your upcoming appointment in genetics? (0 = not at all, 100 = very good) Video Evaluation Understandability: How understandable were the contents of the video for you? (0 = not understandable at all; 100 = very understandable) Helpfulness: How helpful did you find the video you watched? (VAS, 0 = not helpful at all; 100 = very helpful) Amount of information: The amount of information in the video was…? (too much, just right, too little, (single choice item)) [file mmc1.docx]

| B: Video Evaluation Supplementary Table 1. Participants‘ Evaluation of Videos‘ Understandability and Helpfulness | | | | | | | | | | |
| --- | --- | --- | --- | --- | --- | --- | --- | --- | --- | --- |
| **Video** |  | Understandability | | | |  | Helpfulness | | | |
|  | *n* | *Min* | *Max* | *M* | *SD* |  | *Min* | *Max* | *M* | *SD* |
| **1** | 177 | 0 | 100 | 98.01 | 11.42 |  | 0 | 100 | 91.66 | 19.35 |
| **2** | 177 | 0 | 100 | 98.85 | 8.08 |  | 0 | 100 | 95.81 | 14.73 |
| **3** | 153 | 50 | 100 | 97.84 | 7.60 |  | 5 | 100 | 94.93 | 13.97 |
| **4** | 24 | 50 | 100 | 93.75 | 13.51 |  | 0 | 100 | 79.21 | 33.45 |
| **5** | 24 | 50 | 100 | 95.17 | 12.39 |  | 0 | 100 | 81.08 | 34.41 |
|  | | | | | | | | | | |
